# The CYP7A1 gene rs3808607 variant is associated with susceptibility of tuberculosis in Moroccan population

**DOI:** 10.11604/pamj.2014.18.1.3397

**Published:** 2014-05-01

**Authors:** Mounia Qrafli, Youssef Amar, Jamaleddine Bourkadi, Jouda Ben Amor, Ghali Iraki, Youssef Bakri, Saaîd Amzazi, Ouafae Lahlou, Fouad Seghrouchni, Rajae El Aouad, Khalid Sadki

**Affiliations:** 1 Laboratory of Human Genomic, National Institute of Hygiene, Rabat, Morocco; 2 Laboratory of Biochemistry and Immunology, Faculty of Sciences, University Mohammed V, Rabat, Morocco; 3 Department of Pneumophtisiology, Moulay Youssef Hospital, Rabat, Morocco

**Keywords:** Tuberculosis, cholesterol 7-alpha-hydroxylase, polymorphisms, SNPs, cholesterol

## Abstract

**Introduction:**

Despite the medical progress in treatment. Tuberculosis (TB) continues to be a serious global health problem. A genome-wide linkage study identified a major susceptibility locus on chromosomal region 8q12-q13 in Moroccan TB patients. The CYP7A1 gene is located in this region and codes for cholesterol 7a-hydroxylase, an enzyme involved in cholesterol catabolism.

**Methods:**

We selected three SNPs (rs3808607, rs8192875 and rs8192879) and studied their genotype and allele frequencies distribution in patients with pulmonary (PTB) or pleural TB (pTB), and compared them to Healthy Controls (HC). Genotyping of rs8192875 and rs8192879 SNPs was carried out using the Taq Man SNP genotyping Assay while rs3808607 was investigated by PCR-RFLP.

**Results:**

We reported here for the first time a statistically significant increase in the AA homozygote genotype frequency of rs3808607 in PTB patients compared to HC (p = 0.02, OR = 1.93, 95% CI: 1.93 (1.07;3.49). The increased risk of developing TB was maintained when we combined the groups of patients (PTB-pTB) (p = 0.01, OR= 1.91, 95% CI = (1.07 - 3.42). In contrast, no genetic association was observed between the rs8192875 or rs8192879 polymorphisms and TB.

**Conclusion:**

Our investigations suggest that rs3808607 may play a role in susceptibility to TB in a Moroccan population.

## Introduction

Tuberculosis (TB) is one of the oldest diseases to still affect the human race and is the cause of high mortality and morbidity worldwide. According to the seventeenth global report on TB published by the World Health Organization (WHO) the numbers of TB patients will increase in coming decades [1]. It has been estimated that one-third of the world's population is infected by *Mycobacterium tuberculosis (Mtb)*. However, approximately only 10 % develop the disease, which suggests that TB is due to an as yet unde@257;ned risk factor.

Host cholesterol levels have been implicated in the outcome of mycobacterial infections. It has been found that TB patients have a low serum level of cholesterol so hypocholesterolemia was proposed as a risk factor for TB [2]. Cholesterol has been considered for treatment of pulmonary TB since a cholesterol-rich diet significantly accelerated the bacteriological sterilization of sputum of pulmonary tuberculosis patients [3]. Furthermore, it has been observed that the total serum cholesterol, HDL-C and LDL-C correlated with the evaluated radiological extent of the disease and the degree of smear positivity in pulmonary TB patients [4]. Likewise, molecular and biochemical analyses suggested that the development of the human TB granuloma to caseation correlated with pathogen-mediated dysregulation of host lipid metabolism [5]. Several studies have demonstrated the important role played by cholesterol in the function of the immune system [6, 7]. Cholesterol is required for the adequate development of the cytotoxic function of human lymphocytes [6]; it participates in cytotoxic lymphocyte differentiation and proliferation [7]. Furthermore, lipid rafts (LR), plasma membrane microdomain enriched in cholesterol and sphingolipid, are involved in diverse signaling pathways of the immune system. It has been shown that δγ T cells stimulation by *Mtb* antigens induces LR aggregation suggesting contribution of LR to δγ T cells activation [8]. Importantly, in view of the toxicity associated with the accumulation in *Mtb* of some cholesterol metabolites such cholest-4-en-3-one [9], it has been suggested that the cholesterol degradation pathway could be an interesting avenue for the development of new anti-tuberculosis agents [10]. It has been reported that host cholesterol is also involved in *Mtb* infection and persistence [11, 12]. Moreover, several studies have reported the important role played by LR in the pathogenesis of *Mtb* infections [13]. So the implication of cholesterol in determining the fate of TB is still not completely understood. Nevertheless, there is a yet undefined link between TB and cholesterol. The homeostasis of cholesterol is regulated principally by the cholesterol 7-alpha-hydroxylase pathway. The *CYP7A1* gene codes for cholesterol 7-alpha-hydroxylase (CYP7A1), a cytochrome P450 that plays an essential role in maintaining a balance in cholesterol and bile acid since its catalytic reaction is the rate-limiting step and the major step in regulating homeostasis of cholesterol and bile acids [14]. The *CYP7A1* gene spans about 10 kb and contains 6 exons, 5 introns, one 5´-UTR, and one 3´-UTR and is localized on chromosome 8q12.1.

Genetic variations in the *CYP7A1* gene have been associated with metabolic disorders of cholesterol and bile acids, in addition to a risk of developing several diseases. In this context, it has been reported that a polymorphism in the promoter region of *CYP7A1* named -278 A>C (rs3808607), from the translation initiation codon, or -204 A>C from the transcriptional start site were found to be associated with hypercholesterolemia-hyperlipidemia [15–17], a risk of atherosclerosis [18], colorectal cancer [19] and hypertension [20]. In addition, a *CYP7A1* enzyme deficiency caused by a homozygous 1302–1303 delTT deletion mutation in *CYP7A1* exon 6, leading to a frameshift (L413fsX414), has been linked to a hypercholesterolemic phenotype [16]. Importantly, *CYP7A1* gene is located within an interesting chromosomal region, the 8q12-q13 that is associated with pulmonary TB in a Moroccan population [21]. In the present study, we examined the polymorphism of three SNPs located in the *CYP7A1* gene and evaluated their impact on susceptibility to TB of a Moroccan population by using a case-control approach.

## Methods

### Study designs

Three hundred and twenty-four informed and unrelated adult volunteers were recruited for this immunogenetic study during two years, as of 2010. TB patients were represented by 136 PTB patients with sputum positive for acid-fast staining and mycobacterial culture and 50 pTB patients biopsy positive for granulomatous lesions and mycobacterial culture. All patients were tuberculin skin test positive. Patients were recruited from 19 public health centres, including the Centers of TB Treatment and Respiratory Disease (CTRD) and the university hospitals. Healthy controls (HC) included 138 subjects matched for sex and age and were recruited from the Regional Centers of Blood Transfusion (RCBT). All moroccan ethnic groups (Arabs, Sahraoui and Berbers) are equally represented between Patients and healthy subjects.

All HC were tuberculin skin test negative and retained this immunological status during the two years after recruitment to a posterior telephonic check-contact. Ten ml of peripheral blood was collected in ethylenediaminetetraacetic acid tubes from all participants. Structured questionnaires were used to collect all information about demographic parameters and data on the medical history. The observed sex ratio (males /females) for TB patients was almost similar to that of HC (106/30 vs 105/33, respectively). The mean age ± standard deviation (SD) was 33.43 ±13.24 years, ranging from 18 to 67 years, and 32.41 ±11.10 years, ranging from 18 to 61 years, for patients and HC, respectively. All subjects were negatives for HIV-1/2 infection (tested by Axsym Assays, Abbott Laboratories, Chicago, IL, USA). This study was approved by the local ethics committee (Faculty of Medicine and Pharmacy from the Mohammed V-Souissi University) and informed written consent was obtained from all subjects.

### CYP7A1 genotyping

DNA extraction: Total genomic DNA was extracted from peripheral blood of all TB patients and HC using QIAampDNA Blood Maxi kit (QIAamp DNA Blood Mini Kit, Qiagen GmbH, Hilden, Germany). DNA samples were stored at -20°C until use. CYP7A1 selected SNPs: Three functional SNPs of the CYP7A1 gene were selected for genotyping. When considering their position on the gene and the quality of the mutations we supposed that they could have a potential effect on the expression of the CYP7A1 enzyme: the first SNP rs8192875 C>T is located in the fourth exon and it represents a nonsense mutation. The second SNP rs8192879 C>T is in the 3'UTR region, in a linkage disequilibrium block (YRI-B1) [22]. The third SNP rs3808607 A>C is located in the promoter region

Real time PCR genotyping: Two of the three selected SNPs were genotyped by Real time PCR technology for 136 PTB and 138 HC. In fact, rs8192875 and rs8192879 SNP genotyping was performed with a TaqMan^®^ Drug Metabolism Genotyping Assays (Applied Biosystems, Foster City, CA). Reactions were performed as recommended by the manufacturer (Applied Biosystems), in a total volume of 25 μl containing 1.25 μl of Drug metabolism genotyping assay 20 X, 12.5 μl of TaqMan 2 X Universal PCR Master Mix (Applied Biosystems) and 11.25 μl of DNA sample diluted in DNase-free water. Amplification was carried out for 50 cycles at 92°C for 15sec as a denaturation step, followed by an annealing/extension period at 60°C for 90 sec, with an initial denaturation period of 10 min at 95°C. PCR allelic discrimination was performed on a 7500 Fast Real-Time PCR System (Applied Biosystems, Foster City, CA, USA), which measured the specific allele fluorescence of each sample.

PCR-RFLP Genotyping: The A–204C of the CYP7A1 gene was genotyped in 100 PTB, 50 pTB and 100 HC. Genotyping was done by the PCR-BsaI restriction fragment length polymorphism method, using primers 5’-AATGTTTTTCCCAGTTCTCTTTC-3’ (sense) and 5’-AATTAGCCATTTGTTCATTCTATTAG-3’ (antisense), as described previously (Wang et al., 1998). PCR primers were designed to amplify a product of 393 bp. Briefly, the PCR was performed in a reaction mixture of 10 μL containing 0.5 IU of Taq DNA polymerase and 1 μL of template DNA with a concentration of approximately 50–150 ng/μL. Preceded by the initial denaturation at 94°C for 4 min, the amplification was carried out for 30 cycles for 30 sec at 94°C, 30 sec at 53°C and 30 sec at 72°C, with a final extension at 72°C for 7 min. The PCR product of 393 bp was digested in a total volume of 20 μL with 10 IU of BsaI for 3 hr at 50°C. After enzymatic digestion two fragments of 93 bp and 300 bp for the A allele, and three fragments of 39 bp, 93 bp and 261 bp for the C allele were obtained. The digested products were visualized and photographed in 3% agarose gels stained with ethidium bromide. To validate the results obtained by PCR-RFLP, the genotyping of some randomly selected samples of each genotype profile were confirmed by DNA sequencing using an automated sequencing system (ABI Prism 3730 Genetic Analyzer), following the manufacturer's protocols.

### Statistical analysis

The genotype frequencies and allele distribution between patients and HC were estimated by direct counting. The Hardy-Weinberg equilibrium was tested in the first step of statistical analysis. Statistical analysis was carried using EPI INFOTM software, version 7.1.0.6 (Centers for Disease Control and Prevention, Atlanta, GA; 08 September 2012). All results corresponding to p-values below 0.05 were considered statistically significant. Moreover, the odds ratio (OR) with a 95% confidence interval (CI) was calculated to evaluate the risk of association between genotypes or alleles and TB disease.

## Results

All genotype frequencies of patients and HC were consistent with respect to the Hardy-Weinberg equilibrium. In fact, each Khi2 value calculated for each SNPs for both patient and HC groups are inferior to 3.84.

### CYP7A1 promoter polymorphism and risk of TB

The genotype and allele frequency distribution of the *CYP7A1* -204 A>C polymorphism (rs3808607) between PTB and pTB patients and the HC groups are given in Table 1. A significant statistical difference in both the AA genotype and A allele frequencies have been observed between PTB patients and HC (43% vs 28%, p = 0.026, OR = 1.94, 95% CI = 1.03-3.65 and 65% vs 54%, p = 0.025, OR = 1.58, 95% CI =1.04–2.4, respectively). The same tendency in the AA genotype and allele frequencies distribution was found between pTB and HC, differences were not statitical reached (42% vs 28%, p>0,05 and 64% vs 54%, p> 0,05, respectively). Furthermore, We observed a significant increase of homozyote AA genotype and A allele frequencies in combined patient groups (PTB - pTB) compared with HC (43% vs 28%, p =0.018, OR = 1.91, 95% CI= 1.07-3.42 and 65% vs 54%, p = 0.016, OR = 1,56 (1.06-2.28), respectively).

**Table 1:** Genotypes and alleles frequencies for the rs3808607*CYP7A1* polymorphism in TB (TBP, pTB) patients vs HC

Patients vs Controls						
rs3808607	PTB N= 100 (%)	PTB N = 50 (%)	PTB-pTB N = 150 (%)	HC N = 100 (%)	P value	OR (95% CI)
**Genotypes (N)**						
**AA**	43(43)	21 (42)	64 (43)	28(28)	0,026^a^	1,94 [1,03-3,65]^a^
					>0,05^b^, 0.018^c^	1,91 [1,07-3,42]^c^
**AC**	44 (44)	22 (44)	66 (44)	52 (52)	>0,05^a^^,^^b^^,^^c^	
**CC**	13 (13)	7 (14)	20 (13)	20 (20)	> 0,05^a^^,^^b^^,^^c^	
**Alleles (2N)**						
**A**	130 (65)	64 (64)	194 (65)	108 (54)	0,025^a^	1,58 [1,04-2,41]^a^
					>0,05^b^, 0.016^c^	1,56 [1,06-2,28]^c^
**C**	70 (35)	36 (36)	106 (35)	92 (46)	0,025^a^	0,63 [0,41-0,96]^a^
					>0,05^b^,0.016^c^	0,64 [0,44-0,94]^c^

Abbreviations: CI= confidence interval; OR = odds ratio

a PTB vs HC

b pTB vs HC

c PTB-pTB vs HC.

### rs8192879 CYP7A1 SNP genotyping

Statictical analysis of the rs8192879 polymorphism showed no significant difference in the genotype or allele frequency distribution for HC compared to PTB patients (p> 0,05) (Table 2). At allelic level, we observed a high frequency of C allele than T allele in both PTB patients and HC groups (75.72% vs 24.28% and 72.79% vs 27.21%, respectively).

**Table 2:** Genotypes and alleles frequencies for the rs8192879 *CYP7A1* polymorphism in PTB patients and HC

PTB vs HC			
rs8192879	PTB N= 136 (%)	HC N= 138 (%)	P value
**Genotypes (N)**			
**CC**	69 (50,73)	82 (59,42)	>0,05
**CT**	60 (44,11)	45 (32,6)	>0,05
**TT**	7 (5,14)	11 (7,97)	>0,05
**Alleles (2N)**			
**C**	198(72,79)	209(75,72)	>0,05
**T**	74(27,21)	67 (24,28)	>0,05

Abbreviations: PTB = pulmonary tuberculosis; HC = Healthy controls

### rs8192875 CYP7A1 SNP genotyping

The results regarding the *CYP7A1* rs8192875 C>T SNP are shown in Table 3. No homozygous TT or heterozygous CT individuals were observed among neither the PTB patients nor the HC. There was no significant difference in the genotype and allele frequencies distribution between these two groups 100% vs 100% for AA genotype, p > 0,05).

**Table 3:** Genotypes and alleles frequencies for the rs8192875 *CYP7A1* polymorphism in PTB patients and HC

PTB vs HC			
rs8192875	PTB N = 136 (%)	HC N = 138 (%)	P value
**Genotypes (N)**			
**CC**	136 (100)	138(100)	>0,05
**CT**	0 (0)	0 (0)	>0,05
**TT**	0 (0)	0 (0)	>0,05
**Alleles (2N)**			
**C**	272 (100)	276 (100)	>0,05
**T**	0 (0)	0 (0)	>0,05

Abbreviations: PTB = pulmonary tuberculosis; HC = Healthy controls

However, there was no significant difference in the genotype and allele frequencies distributions for each SNP between Moroccan ethnic groups (p-value > 0.05) (data not shown).

## Discussion

On the basis of numerous observations, evidence supports the hypothesis of contribution of host genetic factors in determining of susceptibility/resistance to TB [23, 24]. In this context, a previous reported studied genetic association in a Moroccan population with TB and suggested the potential involvement of some genes in the susceptibility or resistance to TB [25–27].

Here, using a candidate gene approach we investigated the association of three genetic variants in the *CYP7A1* gene with susceptibility to TB disease. To our knowledge the work presented here reported for the first time the relationship between *CYP7A1* gene polymorphism and susceptibility to TB. In fact, to reach this principal objective, we also report for the first time the genotype and allele frequencies distribution in a Moroccan population, which represents a North-African population. Comparison of the allele frequencies of all the SNPs investigated with those reported in HapMap database and in the literature revealed an interesting result: the mutant allele frequency seems to be different for each SNP between Caucasians, Chinese, Nigeria and Japanese populations. Hence, in our population the T allele of rs8192875 C>T could be considered as a rare allele as determined by its allele frequency. This result seems to be similar to that observed in Caucasians and Chinese populations (0% and 1%, respectively) and little different to that reported in Nigerian and Japanese populations (2% and 6%, respectively). In addition, the T allele frequency of rs8192879 C>T SNP reported here (24.28%) was similar to that observed in Nigerian, Japanese and Chinese populations (22%, 26%, 27%, respectively). In contrast, this allele frequency is lower than that reported in a Caucasian population (37%). The lack of association between the rs8192875 and rs8192879 polymorphisms and TB disease may indicate that these variations had no effect on conferring predisposition to TB in our population.

Interestingly, the C allele frequency of rs3808607 SNP found in our study is comparable to that reported in Chinese population (45.2%) [20], and it appears to be relatively higher than that reported in Caucasian (39%) [17] and North Indian populations (39.8%) [28]. This allele may play a protective role since it is negatively associated with TB. Statistical analysis of our data report a significant positive association between the A allele and the AA homozygote genotype of the rs3808607 SNP and TB disease. It seems that AA genotype-carriers had almost a double risk of developing TB disease in our population. Furthermore, in the pTB patient group, the AA homozygote genotype was also found at a higher frequency compared to the HC (42% vs 28%), though this difference failed to reach statistical significance, suggesting a potential contribution of this specificity on susceptibility to pTB disease. The lack of association observed in this case could be attributed to the small size of the pTB patient group.

While we put together all the TB patients into the same group: PTB-pTB, the increase risk of developing TB was still sustained. These finding should be confirmed in a larger population. The genetic association revealed that the promoter region could play an important role in the control of the disease. Earlier studies reported that deletion of the -213 to 91 segment from the *CYP7A1* promoter caused a 40% reduction in the promoter activity suggesting the location of an important regulatory element [29]. The location of this polymorphism in the promoter region could influence the interaction of transcription factors with theirs receptors on this region and consequently could modulate the transcriptional activity of this gene.

Based on the substantial and relevant data concerning on the one hand the function of cholesterol 7 alpha-hydroxylase in regulation of the balance in cholesterol and the crucial role played by cholesterol on the immune system [6, 7] and on the other hand the impact of genetic variations occurring at position -204 at the promoter region of the *CYP7A1* gene [16, 19], we can hypothesize on the development of TB disease in patients carrying an AA genotype of the rs3808607SNP. First of all, the AA genotype of *CYP7A1* was associated with an elevated low-density lipoprotein (LDL) cholesterol concentration in an Inuit Canadian population but not in Hutterites and Oji-Cree Canadian populations [30]. In addition, a significant increase in serum triglycerides in a healthy normolipidaemic male population and with significantly higher concentrations of total cholesterol in hypertriglyceridemic patients AA genotype carriers it was observed in a Netherlands population [17]. In contrast, others studies reported the opposite results, since the LDL-cholesterol concentrations were higher in CC than AA homozygotes both men and women [15]. Differences in the genetic background of the populations may be among the reasons for these discrepancies. Furthermore, the AA genotype was associated with other diseases such as hypertension in obese male patients [20]. De Castros-Orós and colleagues suggested that compared with the A-allele, the C-allele variation was associated with enhanced gene expression [31]. According to this observation we could suppose that the AA genotype is associated with decreased gene expression. The -204A allele may participate in the repression of gene expression by increasing the affinity to negative regulators or in an opposite manner by decreasing the affinity to one or more positive regulators acting on the promoter region of the *CYP7A1* gene,which is linked the cholesterol metabolism to immune system. This hypothesis is supported by Lu and his colleagues from studies into liver X receptors (LXRs) [32]. LXRs are nuclear receptors that play a central role in the transcriptional control of cholesterol homeostasis. They comprise two members LXRa and LXRs. LXRs are also important for the proper control of genes involved in the innate immune response to bacterial pathogens and mice lacking LXRs were more susceptible to *Mtb* infection [33].

Interestingly, in a murine model, it has been reported that LXRa stimulates the expression of *CYP7A1* via binding to a potent LXR response element (LXRE) present in the *CYP7A1* promoter [34]. Additionally, it has been shown that LXRa with LHR-1 can act as a positive gene expression regulator of *CYP7A1* in mice [32]. So, we can hypothesize that any genetic variation occurring in this region of the *CYP7A1* promoter (LXRE) could theoretically influence *CYP7A1* gene expression. The *CYP7A1* will be repressed and consequently the cholesterol will be more available to pathogens. As cholesterol is required by *Mtb* for infection and persistence, it acts as a virulence factor [11, 12]. So, it seems that the AA homozygous subjects were more susceptible to develop TB disease.

Finally, we cannot exclude the possibility that the -204 A allele is in linkage disequilibrium with a yet unidentified functional polymorphism at the *CYP7A1* gene itself or other closely located genes directly or indirectly related to immune system pathways implicated in defense against TB. More recently, Grant and colleagues have identified a statistically significant association between two genetic variants (rs2726600 and rs1568952), located within the TOX gene and PTB disease in a Moroccan population [35]. The *CYP7A1* gene is located close to the TOX gene and it would be interesting to do a statistical haplotype analysis to evaluate the risk of each variant on the susceptibility in the Moroccan population.

## Conclusion

In summary, we identified for the first time a significant association of the A-204C polymorphism of the *CYP7A1* gene and development of TB in a Moroccan population. This finding should be confirmed on a larger sample size. In addition, further studies should give a better understanding of the involvement of *CYP7A1* in terms of the susceptibility to TB.
